# Long-Term Efficacy and Safety of 0.1% Cyclosporine A Cationic Emulsion in Advanced Dry Eye Disease: A 24-Month Retrospective Study

**DOI:** 10.3390/ph18111682

**Published:** 2025-11-06

**Authors:** Monika Sarnat-Kucharczyk, Martyna Nowak, Ewa Mrukwa-Kominek

**Affiliations:** 1 Department of Ophthalmology, Faculty of Medical Sciences in Katowice, Medical University of Silesia, 40-514 Katowice, Poland; emrowka@poczta.onet.pl; 2 Kornel Gibinski University Clinical Centre, 40-514 Katowice, Poland; martyna.jacak@gmail.com

**Keywords:** dry eye disease, cyclosporin, ocular surface disease index, tear break-up time, corneal staining (Oxford scale), Schirmer test

## Abstract

**Background**: To evaluate the effectiveness of 0.1% cyclosporine A (CsA) cationic emulsion in managing advanced dry eye disease (DED), based on clinical parameters: Ocular Surface Disease Index (OSDI), best-corrected visual acuity (BCVA), Tear Break-Up Time (TBUT), corneal fluorescein staining (CFS) on the Oxford scale, Schirmer test, and intraocular pressure (IOP). **Methods**: This retrospective study included 20 patients (40 eyes) with severe DED unresponsive to previous therapies. All patients continued artificial tears and added 0.1% CsA once daily. Baseline assessments included OSDI, BCVA, TBUT, corneal staining, Schirmer test, and IOP. Follow-ups occurred at 1–3, 6, 12, and 24 months. Data were analyzed for treatment effect and progression over time. **Results**: The mean age was 53.5 ± 13.5 years; 80% were female. BCVA showed no significant changes. OSDI scores improved from severe (>53) to moderate (approximately 35). Schirmer test increased from ~6.2 mm to >10 mm (*p* < 0.001). TBUT improved from approximately 6 to 10 s (*p* < 0.001), with significant differences after 6 months. CFS scores decreased from 3.4 to 2.05 (*p* < 0.001), indicating reduced corneal damage. IOP remained stable throughout the study period. **Conclusions**: Long-term use of 0.1% cyclosporine A cationic emulsion led to marked and sustained improvement in both subjective symptoms and objective ocular surface parameters in severe dry eye disease. The therapy was safe, well tolerated, and did not affect visual acuity or intraocular pressure, supporting its value as a long-term treatment option.

## 1. Introduction

Dry eye disease (DED is described in the latest Dry Eye Workshop III (DEWS III) report by the Tear Film and Ocular Surface Society (TFOS) as a complex, multifactorial disorder of the ocular surface [1]. This condition is defined by a disturbance in the homeostasis of the tear film, presenting with core features such as tear film instability, hyperosmolarity, ocular surface inflammation, tissue damage, and neurosensory abnormalities [2]. These etiological factors contribute to a frequent discordance between clinical signs and patient-reported symptoms [3,4].

The global prevalence of DED varies widely, reflecting inconsistent diagnostic criteria and the complex nature of the disease. Prevalence estimates range from 5% to 33% in population studies, while patient-reported symptoms can raise estimates up to 75% in some cohorts. DED is more common in women and becomes more prevalent with age [5,6,7].

DED leads to a variety of ocular surface alterations, including corneal epithelial damage, inflammation, and nerve dysfunction, which may further impair tear film stability and visual quality [8,9].

Treatment for DED is conducted in phases, with therapeutic options adjusted based on the condition’s underlying causes and severity [10]. The pace of treatment escalation should reflect the disease’s progression [11]. If a patient doesn’t respond to first-line treatments such as artificial tears or lifestyle modifications, second-line therapies—like topical corticosteroids, cyclosporine, or lifitegrast—are typically recommended.

Early-stage interventions rely on low-risk, widely accessible options such as patient education, eye hygiene, and non-prescription products. In more advanced cases, treatment should focus immediately on the specific underlying issues, like tear production deficiency or meibomian gland dysfunction, using options such as anti-inflammatory agents such as corticosteroids and topical cyclosporine A (CsA), surgical methods, dietary adjustments, and complementary therapies [12].

In 2003, a 0.05% anionic oil-in-water CsA emulsion was approved by the U.S. Food and Drug Administration to stimulate tear production in individuals diagnosed with keratoconjunctivitis sicca. Due to the lipophilic nature of CsA, an optimized formulation was subsequently developed as a cationic emulsion (CE) with preservative-free 0.1% (1 mg/mL) CsA, designed to enhance ocular surface residence time and bioavailability. In 2015, this CsA CE formulation gained regulatory approval in the European Union for the treatment of severe keratitis in adult DED patients refractory to tear substitute therapies [13]. CsA is now widely used for moderate-to-severe DED [14,15], with efficacy based on corneal/conjunctival staining, poor corticosteroid response, or persistent inflammation [16,17]. To improve comfort and adherence, a short course of corticosteroids may be used before initiating CsA, reducing initial drop-related irritation and expediting symptom relief [18,19,20]. Corticosteroids are used for a limited duration due to potential side effects, and patients should be continuously monitored during therapy [20,21,22].

In recent years, CsA has gained increasing popularity for treating moderate to severe DED symptoms [14,15]. Unlike corticosteroids, it can be administered safely over extended periods without significant side effects [16].

CsA is recommended to be used to alleviate inflammation in DED, with the decision to initiate treatment based on factors such as the degree of corneal and conjunctival staining, the rapidity of response to corticosteroid therapy, and lack of improvement with standard treatment.

A lack of corneal staining should not, however, exclude CsA as a therapeutic option for controlling inflammation if other risk factors are present. Patients exhibiting clinical risk factors for developing acute DED may benefit from early-stage CsA treatment when symptoms first appear [17].

Long-term use of CsA can lead to a reduction or even complete remission of DED symptoms, allowing patients to consider dose reduction or discontinuation (although there is a risk of recurrence). Markers of inflammation—such as conjunctival and corneal staining, tear film osmolarity, and conjunctival hyperemia—demonstrate the strong anti-inflammatory effectiveness of CsA. Patients generally exhibit high tolerance to the medication, with minimal side effects, and systemic adverse reactions are rare [13].

The recently published TFOS DEWS III Management and Therapy Report provides a comprehensive review of over 1000 studies on dry eye disease (DED) treatment strategies. It highlights advances in tear supplementation, including lipid-based and ectoine-containing formulations, as well as therapies targeting meibomian gland dysfunction such as intense pulsed light (IPL), thermal devices and plasma therapy. Anti-inflammatory agents and biologic tear substitutes like autologous serum and platelet-rich plasma are also emphasized. Novel approaches such as intranasal neuromodulation, salivary gland transplantation and lacrimal gland reinnervation are discussed alongside the importance of lifestyle modifications including diet, blinking habits, environmental control and lid hygiene [3].

**Figure 1:**
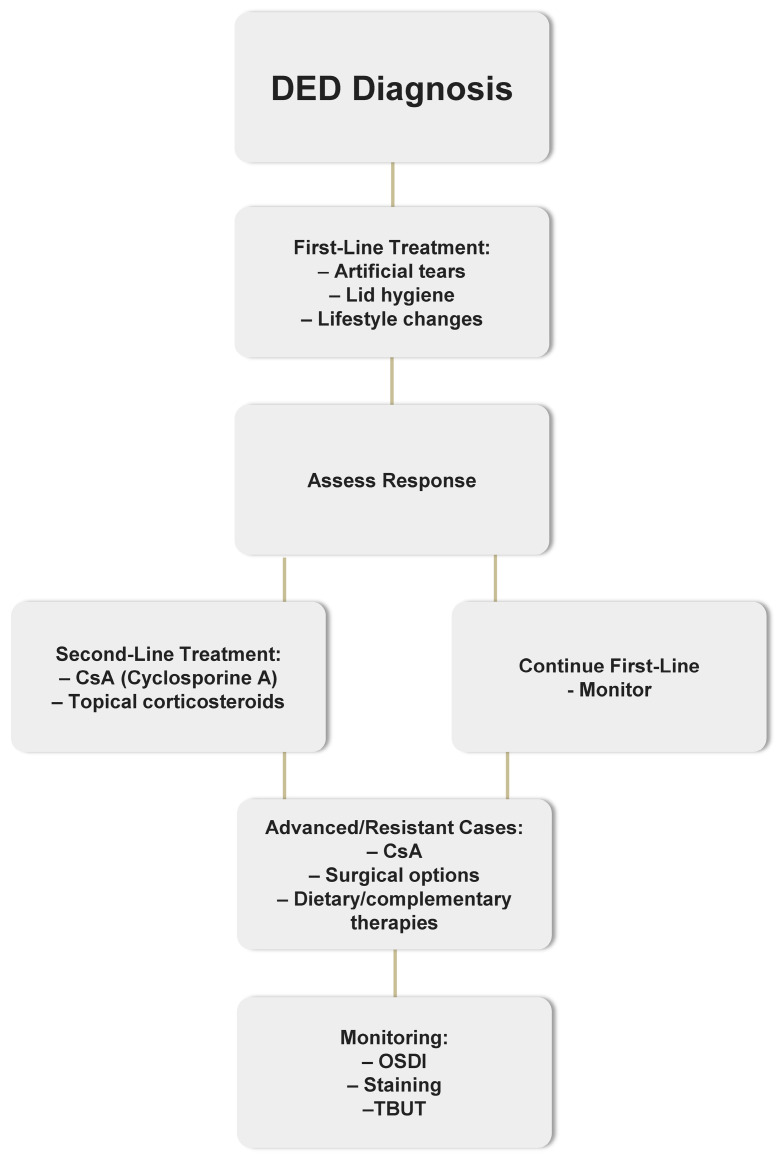
Stepwise management algorithm for dry eye disease (DED). Abbreviations: DED—dry eye disease; CsA—cyclosporine A; OSDI—Ocular Surface Disease Index; TBUT—tear break-up time.

The management of Dry Eye Disease (DED) follows a structured, stepwise approach based on disease severity and treatment response. A simplified flowchart outlining this therapeutic pathway is presented in Figure 1.

The objective of this retrospective study is to evaluate the long-term efficacy of 0.1% CsA cationic emulsion (eye drops, 1 mg/mL) in patients with chronic, severe DED. To this end, the study assesses changes in best-corrected visual acuity (BCVA), OSDI, tear film aqueous secretion via the Schirmer test, tear film breakup time (TBUT), anterior ocular surface damage using corneal fluorescein staining (CFS) graded with the Oxford scale, and intraocular pressure (IOP), all during ongoing CsA therapy.

## 2. Results

### 2.1. Patient Demographics

Among the 20 patients included in the study, 80% were women, which is consistent with the known higher prevalence of severe dry eye disease (DED) in females. The mean age at the initiation of 0.1% CsA therapy was 53.5 ± 13.5 years (range: 24–70).

At baseline, the mean OSDI score was 53.1 ± 13.8, indicating severe symptoms (OSDI > 33). The mean Oxford scale score averaged 3.4 ± 0.9, reflecting moderate to severe ocular surface damage.

Tear film parameters were also impaired: the mean TBUT was 5.95 ± 2.9 s and Schirmer test results were 6.2 ± 2.2 mm/5 min in the right eye and 6.25 ± 2.8 mm/5 min in the left, confirming reduced tear secretion. Baseline BCVA and intraocular pressure (IOP) were within normal ranges and did not differ significantly between eyes (*p* > 0.05).

Baseline characteristics are summarized in Table 1.

### 2.2. OSDI

A repeated-measures ANOVA for OSDI scores revealed a statistically significant improvement over time. Since the sphericity assumption was violated, Greenhouse–Geisser correction was applied. The initial mean OSDI score was 53.13, decreasing to 35.65 after 24 months of CsA treatment. Significant changes were observed from 6 months onward (F(2.82, 53.63) = 46.41, *p* < 0.001, η^2^p = 0.71, ε = 0.71, Greenhouse–Geisser-corrected). Bonferroni-adjusted post hoc tests showed significant reductions in symptom scores from baseline (mean = 49.93) at month 6 (mean = 39.86, *p* = 0.00005), month 12 (mean = 36.00, *p* < 0.001), and month 24 (mean = 34.71, *p* < 0.001). No significant difference was observed between months 12 and 24 (*p* = 1.000).

**Figure 2:**
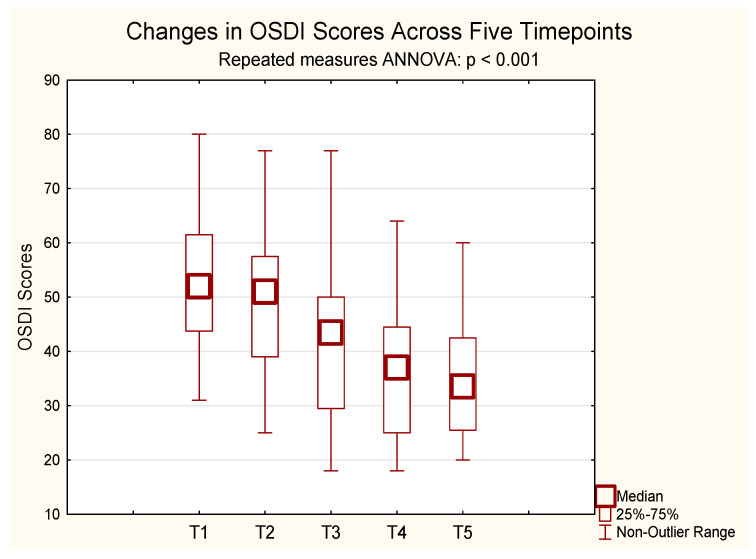
Mean OSDI scores over five time points. Error bars represent standard deviation. A statistically significant reduction in OSDI was observed across time (*p* < 0.001, repeated-measures ANOVA, *n* = 20). T1–T5: Consecutive measurement timepoints. T1—baseline; T2—between 1–3 months; T3—at 6 months; T4—at 12 months; T5—at 24 months.

These findings indicate that ocular surface disorder severity decreased progressively over the course of treatment (Figure 2).

### 2.3. BCVA

BCVA values were compared across five time points for both eyes. For the right eye, mean ranks across the five assessments were 2.85 (baseline), 2.88 (1–3 months post-CsA), 3.18 (6 months), 3.25 (12 months), and 2.85 (24 months). For the left eye, mean ranks were 2.75 (baseline), 3.10, 3.05, 3.20, and 2.90, respectively.

No statistically significant differences in BCVA were observed over time for either eye (right eye: χ^2^(4) = 3.24, *p* = 0.519; left eye: χ^2^(4) = 2.72, *p* = 0.606), as determined using the Friedman test, indicating stable visual acuity throughout the 24-month treatment period (Table 2).

### 2.4. TBUT

**Figure 3:**
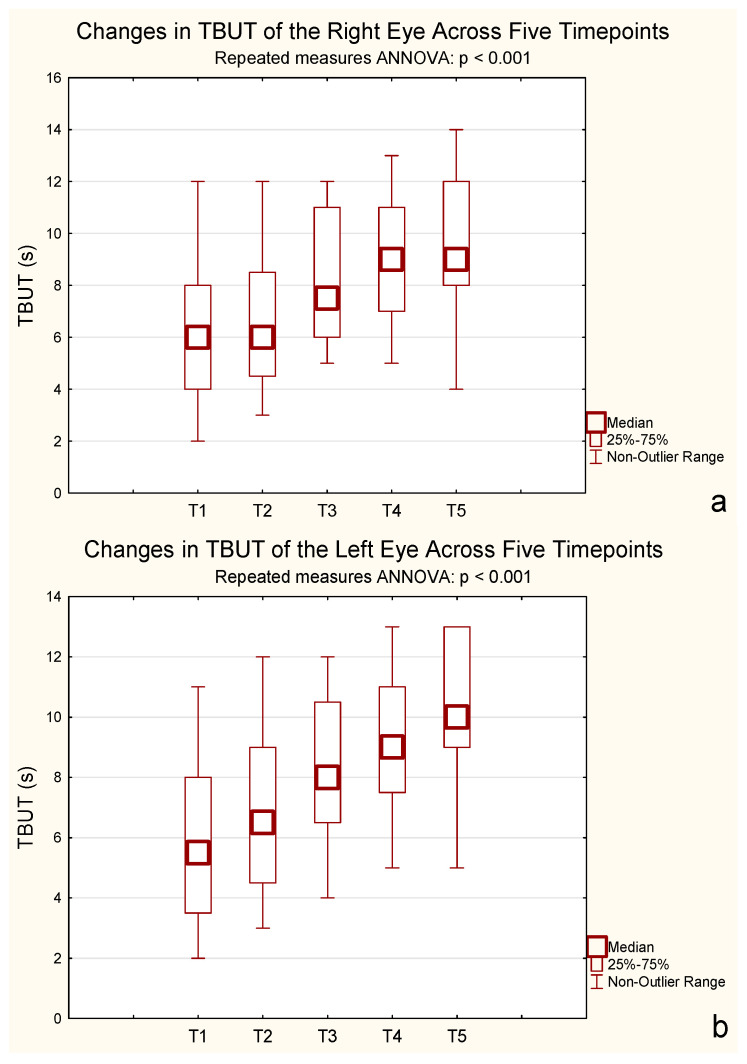
Mean tear film break-up time (TBUT, seconds) over five time points. (**a**) Right eye; (**b**) Left eye. Error bars represent standard deviation. A statistically significant increase in OSDI was observed across time (*p* < 0.001, repeated-measures ANOVA, *n* = 20). T1–T5: Consecutive measurement timepoints. T1—baseline; T2—between 1–3 months; T3—at 6 months; T4—at 12 months; T5—at 24 months.

A repeated-measures ANOVA for TBUT revealed a significant increase over time for both eyes. For the right eye, mean TBUT increased from 5.95 s (SD = 2.93) at baseline to 9.90 s (SD = 2.67) after 24 months, with significant differences observed from 6 months onward (F(2.56, 48.65) = 59.19, *p* < 0.001, η^2^p = 0.76, ε = 0.64, Greenhouse–Geisser corrected). For the left eye, mean TBUT improved from 5.95 s (SD = 2.78) to 10.15 s (SD = 2.39) over the same period, also showing a significant effect (F(2.77, 52.69) = 37.40, *p* < 0.001, η^2^p = 0.66, ε = 0.69, Greenhouse–Geisser corrected). Bonferroni-adjusted post hoc tests confirmed that treatment had a strong effect on tear film stability, particularly from 6 months onward. These results indicate that the treatment significantly extended TBUT in both eyes (Figure 3).

Bonferroni post hoc tests confirmed significant increases from baseline starting at 6 months, with no further differences between 12 and 24 months (*p* = 0.32 and *p* = 1.000, respectively).

### 2.5. CFS on Oxford Scale

Repeated-measures ANOVA with Greenhouse–Geisser correction revealed a significant reduction in Oxford Scale scores, indicating decreased ocular surface damage following CsA treatment (F(2.62, 49.73) = 41.96, *p* < 0.001, ε = 0.65). Mean scores decreased from 3.40 at baseline to 2.05 after 24 months. Bonferroni-adjusted post hoc comparisons revealed significant differences across most time points, confirming the long-term efficacy of the treatment.

### 2.6. Schirmer Test

A repeated-measures ANOVA for the Schirmer test revealed significant improvements over time in both eyes. For the right eye, the mean Schirmer score was 6.20 mm at baseline and gradually increased to 10.20 mm by 24 months, with notable improvements observed after the second measurement point. Similarly, for the left eye, scores rose from 6.25 mm at the start to 10.25 mm, with a meaningful increase observed between the second and third measurements.

The ANOVA results confirmed significant differences across time points for both eyes. In the right eye, F(2.35, 44.57) = 64.65, *p* < 0.001, η^2^p = 0.77, indicating a strong effect size. The assumption of sphericity was violated (ε = 0.59); therefore, Greenhouse–Geisser correction was applied. A repeated-measures ANOVA revealed a significant effect of time on Schirmer test results in the left eye, F(2.76, 52.42) = 52.67, *p* < 0.001, η^2^p = 0.73, ε = 0.69 (Greenhouse–Geisser corrected).

Post hoc Bonferroni tests revealed that tear production significantly improved from baseline (6.00 mm) at 6 months (7.14 mm, *p* = 0.00003), 12 months (8.71 mm, *p* < 0.001), and 24 months (9.71 mm, *p* < 0.001). No significant difference was observed between 12 and 24 months (*p* = 0.265), indicating a plateau in improvement.

**Figure 4:**
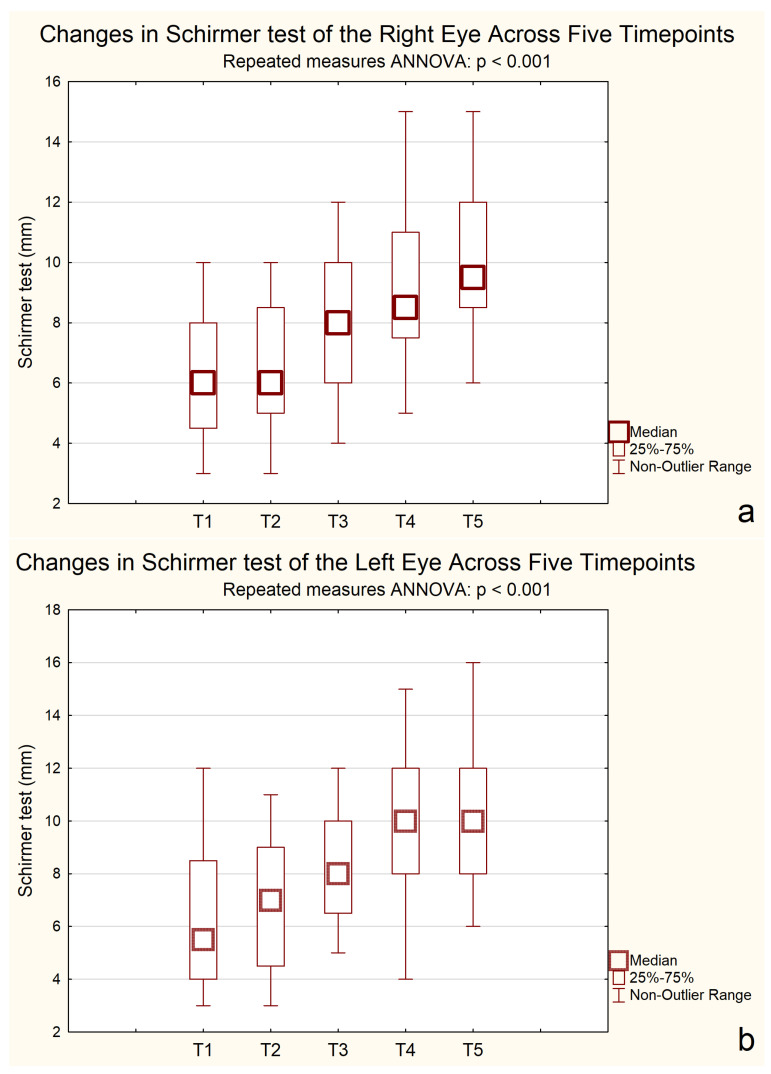
Mean Schirmer test over five time points. (**a**) Right eye; (**b**) Left eye. Error bars represent standard deviation. A statistically significant improvement in Schirmer test results was observed across time (*p* < 0.001, repeated-measures ANOVA, *n* = 20). T1–T5: Consecutive measurement timepoints. T1—baseline; T2—between 1–3 months; T3—at 6 months; T4—at 12 months; T5—at 24 months.

Bonferroni-adjusted post hoc comparisons showed consistent improvements in Schirmer test results, as shown in Figure 4.

### 2.7. IOP

IOP differences were analyzed using repeated-measures ANOVA with Greenhouse–Geisser correction applied separately for each eye.

In the right eye, mean IOP ranged from 16.75 mmHg (SD = 3.23) at baseline to 16.90 mmHg (SD = 3.48) after 24 months. In the left eye, values ranged from 16.65 mmHg (SD = 3.17) to 16.55 mmHg (SD = 3.22) over the same period. ANOVA with Greenhouse–Geisser correction results were not statistically significant (right eye: F(2.91, 55.31) = 0.56, *p* = 0.637 ε = 0.73; left eye: F(2.71, 51.58) = 0.31, *p* = 0.802, ε = 0.68), indicating that IOP remained stable throughout the treatment period (Table 3).

The treatment was well tolerated. Mild transient discomfort, such as burning or redness upon instillation, was reported by some patients; however, no participant discontinued therapy due to adverse effects.

## 3. Discussion

This study demonstrated that long-term topical treatment with 0.1% cyclosporine A cationic emulsion resulted in significant and sustained improvements in both subjective and objective measures of severe dry eye disease. Marked improvement was observed in symptom severity (OSDI), tear production (Schirmer test), tear film stability (TBUT), and ocular surface integrity (Oxford scale). Visual acuity and intraocular pressure remained stable, confirming the favorable safety profile of the therapy.

A gradual but significant improvement was observed in subjective symptoms, as reflected by the Ocular Surface Disease Index (OSDI). The greatest changes occurred between 6 and 12 months, demonstrating the delayed but progressive therapeutic response characteristic of CsA. Baseline OSDI scores indicated severe disease, yet by 24 months a meaningful reduction of over 17 points was recorded, reflecting a clinically significant improvement in patients’ quality of life. Similar findings were reported in the SANSIKA trial, where mean OSDI improvement reached 15–20 points after six months of therapy [13]. The present results suggest a comparable or slightly stronger effect, particularly in those with more severe baseline disease. In middle-aged women (40–60 years), symptoms improved within the first 6 months and remained stable thereafter, confirming a rapid and durable response consistent with earlier DED research [23].

Objective tear parameters showed parallel improvement. Schirmer test results increased gradually throughout the study, with significant enhancement of tear secretion after six months. Comparable findings were reported by Baudouin et al. [24] and in the SANSIKA study [13]. though the effect size in our cohort was larger (η^2^ = 0.84–0.90). The stronger response in women aged 40–60 may be related to hormonal and physiological factors associated with menopause, which influence lacrimal gland function and ocular surface homeostasis [25,26]. These results highlight the potential for age- and sex-specific differences in treatment responsiveness.

Tear film stability, measured by tear break-up time (TBUT), improved significantly in both eyes, with the most marked effects within the first 6 months of therapy. Thereafter, values stabilized, suggesting an early therapeutic ceiling. This temporal pattern aligns with previous reports indicating that maximal benefit of CsA occurs within 6–12 months [23,27]. Earlier studies such as SICCANOVE and the Perspective Group trial demonstrated smaller TBUT improvements [24,28], possibly due to shorter follow-up durations or inclusion of less severe cases. The more pronounced response observed here may reflect both the chronicity and intensity of inflammation in advanced DED, where CsA immunomodulatory action exerts stronger restorative effects.

The analysis of corneal fluorescein staining (Oxford scale) confirmed a progressive improvement in ocular surface integrity following long-term cyclosporine A (CsA) treatment. These findings are in line with previous clinical trials, which demonstrated that CsA promotes epithelial healing by suppressing inflammatory mediators and restoring goblet cell density [13].

In the present study, the improvement became more evident after several months of therapy, which is consistent with the delayed structural recovery of the corneal epithelium observed in earlier reports. While parameters such as TBUT and OSDI tend to respond within the first weeks of treatment, epithelial repair typically requires a longer duration of therapy. This gradual improvement supports the concept that CsA exerts both anti-inflammatory and regenerative effects that accumulate over time [29].

Comparative data from larger clinical trials, such as the SANSIKA study and the work of Geerling et al., confirm that the extent of improvement in CFS increases steadily with treatment duration, particularly after six months of continuous use [28]. The favorable trend observed in our cohort reinforces the efficacy of CsA even in patients with advanced forms of dry eye disease [13]. The effect appeared especially marked in middle-aged and postmenopausal women, suggesting a potential influence of hormonal status on the therapeutic response to CsA. The degree and timing of improvement in corneal staining may therefore depend not only on treatment duration but also on patient-specific factors such as age and baseline disease severity.

Recent randomized phase III trials and real-world studies support the efficacy and safety of 0.1% cyclosporine A formulations in moderate-to-severe dry eye disease (DED). For instance, the ESSENCE-2 phase III trial demonstrated early therapeutic effects, with 71.6% of patients classified as responders after treatment with a water-free 0.1% CsA formulation [30]. Another randomized phase III trial conducted in China also showed significant improvement in corneal fluorescein staining after 29 days of treatment with 0.1% CsA compared with vehicle control [31]. In addition, recent real-world studies with follow-up up to 12 months have confirmed the long-term benefits and tolerability of CsA 0.1% cationic emulsion in patients with mild to moderate and severe DED [32].

Our findings are consistent with these studies but extend the evidence by demonstrating sustained improvement in a cohort of patients with severe DED refractory to previous therapies.

Intraocular pressure (IOP) remained unchanged throughout the 24-month observation, confirming the ocular safety of prolonged CsA use. The absence of any IOP elevation is clinically relevant, as it supports CsA’s favorable safety profile and lack of association with secondary glaucoma [33].

Treatment tolerance was excellent. No serious adverse events occurred, and all patients completed the full study period. Mild transient irritation was reported in a few cases, but no discontinuations were attributed to drug intolerance. In contrast, previous large-scale studies reported withdrawal rates ranging from 6–12% due to adverse events [13,28,29]. The lower rate observed in our cohort may reflect effective patient education about the drug’s mechanism and delayed onset of action. Providing patients with realistic expectations and emphasizing the importance of adherence is critical for achieving optimal therapeutic outcomes [13,29].

These findings support the initial assumption that CsA is effective in patients with more advanced stages of DED, as improvements were more pronounced in this study group compared to previous trials involving less severe cases. The therapy appears particularly beneficial in middle-aged women, where hormonal factors may predispose to tear film instability and inflammatory dysfunction. The results also highlight the importance of patient education in ensuring adherence, particularly given the delayed onset of therapeutic effect and the initial discomfort associated with CsA application. In the broader clinical context, the data suggest that with proper instruction and persistence, long-term CsA therapy can be both effective and well tolerated.

This study has several limitations that should be acknowledged. First, its retrospective design inherently carries a risk of selection bias, as patient inclusion was based on the availability of complete medical records rather than randomized enrollment. Second, the study was conducted at a single clinical center, which may limit the generalizability of the findings to broader patient populations or different clinical settings.

The relatively small sample size may have limited the statistical power of the analysis; however, the consistent trends across multiple objective and subjective measures support the robustness of the observed effects. Furthermore, 80% of the study participants were female, reflecting the higher prevalence of dry eye disease among women. This sex imbalance may limit the generalizability of the findings to male patients, as the small proportion of men prevented a reliable sex-stratified analysis.

Future research should focus on prospective, controlled studies in patients with severe DED to further validate these observations. It may also be valuable to assess the impact of structured patient education on treatment adherence and long-term outcomes in CsA therapy.

## 4. Materials and Methods

### 4.1. Study Design and Setting

This retrospective, single-center, observational study included 20 patients (40 eyes) treated for severe DED at the Ophthalmology Outpatient Clinic of the Kornel Gibinski University Clinical Center, Medical University of Silesia in Katowice. Patients selected for retrospective analysis had received 0.1% CsA cationic emulsion therapy, branded as Ikervis^®^ following unsuccessful prior symptomatic treatment for severe DED.

A retrospective design was chosen to evaluate real-world, long-term treatment outcomes in a homogeneous group of patients with advanced DED, a population that is frequently underrepresented in randomized controlled trials due to disease severity or comorbidities. This approach allows for the assessment of therapeutic efficacy and safety over an extended period under routine clinical conditions.

According to the guidelines of the Bioethics Committee of the Medical University of Silesia, retrospective data analysis is not classified as a medical experiment and therefore does not require ethical approval. All procedures adhered to the principles of the Declaration of Helsinki.

### 4.2. Description of the Treatment

The medication contains 1 mg of CsA per 1 mL of emulsion, along with the excipients, including 0.05 mL of cetalkonium chloride, medium-chain triglycerides, glycerol, tyloxapol, poloxamer 188, sodium hydroxide, and water. According to the manufacturer’s recommendations, this medication is indicated for the treatment of severe keratitis associated with DED in adult patients unresponsive to artificial tear substitutes.

All participants were informed about the possible transient discomfort (burning or redness) associated with CsA instillation prior to treatment initiation. Tolerability and adverse effects were monitored throughout the study period.

### 4.3. Study Group

The diagnosis of dry eye disease (DED) was established according to the TFOS DEWS II criteria, which include patient-reported symptoms (OSDI ≥ 13) in combination with at least one sign of tear film instability (TBUT ≤ 10 s), ocular surface staining, or reduced tear production (Schirmer ≤ 10 mm/5 min) [34].

All patients had previously undergone unsuccessful treatment with various therapeutic approaches, including artificial tears, anti-inflammatory agents, and antibiotics. Despite these interventions, they continued to experience severe symptoms such as burning, itching, and redness. Due to the limited effectiveness of prior treatments, this retrospective analysis aimed to evaluate the impact of 0.1% CsA cationic emulsion on dry eye symptoms.

All 20 patients completed the 24-month follow-up, and no data were missing or excluded from the analysis.

### 4.4. Inclusion and Exclusion Criteria

This retrospective analysis included data from adult patients (aged over 18 years) of both genders who had been diagnosed with severe DED. Eligible patients had previously received artificial tear preparations without satisfactory improvement prior to initiating therapy with 0.1% CsA cationic emulsion. Inclusion criteria, as documented in medical records, required a fluorescein staining score of grade II or higher on the Oxford scale and an OSDI score of at least 30 at baseline.

Patients were excluded if records indicated a history of corneal transplantation, active corneal infections (viral, bacterial, amoebic, or fungal), progressive pterygium, penetrating ocular trauma, or unstable/abnormal intraocular pressure.

### 4.5. Data Collection and Follow-Up

Data were collected at five standardized time points to ensure consistency of observation and allow for longitudinal comparison. Baseline data (measurement 1) were collected before initiating CsA therapy. The study group was monitored starting from baseline (measurement 1 at 0 months), followed by subsequent assessments at 1–3 months (measurement 2), 6 months (measurement 3), 12 months (measurement 4) and finally at 24 months (measurement 5).

Eligible patients were instructed to maintain their current preservative-free artificial tear regimen and supplement it with CsA eye drops. CsA was recommended for application to both eyes once daily in the evening.

The analysis focused on changes in symptoms observed or reported by patients at each follow-up. Throughout the observation period, patients were thoroughly evaluated before and after CsA treatment to assess symptom changes over time.

In this retrospective study, patients considered for CsA therapy completed in-depth interviews and underwent both standard and detailed ophthalmologic exams. Whether patients displayed symptoms or not was key to shaping the direction and design of the study.

From the medical records of the Adult Ophthalmology Outpatient Clinic, the following anonymized data were collected: patient demographics, including gender and age, as well as information on current and past medical conditions, recent surgical procedures, pharmacological treatments, diet, use of stimulants, living and working conditions, and contact lens usage. Family medical history was reviewed, with particular attention to relevant ocular or systemic conditions.

A complete ophthalmic evaluation was performed for each patient. The sequence of ophthalmic assessments was consistent with the current recommendations of the Polish Ophthalmological Society for the diagnosis and management of dry eye disease. These guidelines were developed based on the TFOS DEWS II (Tear Film & Ocular Surface Society Dry Eye Workshop II) report published in 2017 [34].

The sequence of procedures was as follows: first, OSDI scores were obtained to determine the severity of ocular surface disease, and BCVA was measured using Snellen charts. Tear film stability was evaluated using the TBUT test. Corneal staining with fluorescein was performed, and the results were classified according to the Oxford grading scale. Tear production was assessed using Schirmer strips without anesthesia. Finally, intraocular pressure was measured using a Goldmann applanation tonometer (CSO, Florence, Italy).

Anterior segment evaluation was performed using a slit-lamp biomicroscope (Haag-Streit 900.7.3 1527; Koeniz, Switzerland) to examine eyelid margins, palpebral and bulbar conjunctiva, and the cornea. Fundus examination was also carried out using the same slit-lamp system.

All measurements were performed by the same ophthalmologist using standardized diagnostic techniques and consistent testing conditions to minimize inter-observer variability.

### 4.6. Characteristics of Conducted Examinations

#### 4.6.1. Ocular Surface Disease Index (OSDI)

During the study, each patient completed the OSDI questionnaire, which was created to diagnose and assess the severity of DED. Based on it, the impact of the disease can be assessed on the patient’s ability to function and comfort, as well as monitor changes between subsequent examinations.

The questionnaire consists of 12 questions, included in three subscales, 4 of which concern subjective symptoms, 4 assess their impact on the quality of vision, while the remaining ones include environmental factors that can both trigger and exacerbate dry eye symptoms.

OSDI is characterized by high sensitivity and specificity and is considered the most reliable indicator of the condition of the eye surface. The OSDI is assessed on a scale from 0 to 100, with the higher the value, the greater the degree of ocular surface disorders [35,36].

#### 4.6.2. Best-Corrected Visual Acuity (BCVA)

This test was performed monocularly using standard Snellen charts at a distance of 5 m under uniform illumination conditions. The results were converted to decimal notation for statistical analysis.

#### 4.6.3. Tear Break-Up Time (TBUT)

The slit-lamp biomicroscopy examination is a non-invasive, safe, and essential procedure in ocular surface assessment. It combines a microscope with a high-intensity light source to provide magnification and stereoscopic vision, enabling detailed, three-dimensional visualization of the anterior segment of the eye. In the context of dry eye diagnostics, the slit lamp is particularly useful for performing the TBUT test. This test involves the instillation of fluorescein dye into the conjunctival sac, followed by observation under cobalt blue light to measure the time interval between a complete blink and the first appearance of tear film disruption.

#### 4.6.4. Corneal Fluorescein Staining (CFS) Assessed Using the Oxford Scale

The Oxford fluorescein staining system is commonly used in the diagnosis of DED as it is both simple and non-invasive test. During the test, drops of sodium fluorescein solution are applied to the patient’s conjunctival sac, and the patient blinks to spread the solution evenly over the eye surface. Using a slit lamp, the cornea is then examined for any stained areas.

#### 4.6.5. Schirmer Test

The Schirmer test is designed to assess tear production; the rate at which the test strip becomes wet is proportional to the rate of tear production. A specialized, slightly folded filter paper strip marked at 1 mm intervals is placed in the conjunctival sac near the outer corner of the eye, about one-third along the eyelid’s length. The strip remains in place for 5 min, after which the length of wetting is measured. During the test, the patient is instructed to look straight ahead, avoiding blinking, tightly closing the eyes, or keeping them overly open.

#### 4.6.6. Intraocular Pressure (IOP)

Intraocular pressure (IOP) was measured using the Goldmann applanation tonometry method at the slit lamp. Prior to measurement, a fluorescein dye was instilled onto the ocular surface to facilitate visualization and ensure accurate readings.

### 4.7. Statistical Analysis

Statistical analyses were conducted using IBM SPSS Statistics version 25 to address the research questions and test the hypotheses. Descriptive statistics, including mean, standard deviation, minimum, maximum, and quartiles, were calculated for each quantitative variable to characterize the data.

The Shapiro–Wilk test assessed data normality, with a significance level of α = 0.05. The Friedman test, a non-parametric alternative to repeated-measures ANOVA, was used to compare median ranks across groups. Kendall’s W was calculated to determine the effect size. Repeated-measures ANOVA was applied to evaluate differences between groups over time for a single dependent variable, followed by Bonferroni-adjusted post hoc comparisons where applicable.

Analyses were performed separately for the right and left eyes (*n* = 20 for each), with each participant contributing one measurement per eye.

All analyses aimed to confirm the hypotheses, with significance defined at *p* < 0.05.

## 5. Conclusions

Long-term treatment with 0.1% CsA cationic emulsion resulted in significant and sustained improvement in both subjective symptoms and objective ocular surface parameters in patients with severe dry eye disease. After 24 months of therapy, mean OSDI scores decreased by over 17 points, while Schirmer test and TBUT values nearly doubled, indicating enhanced tear secretion and film stability. Corneal staining also improved substantially, confirming the regenerative effect on the ocular surface. Visual acuity and intraocular pressure remained stable, supporting the long-term safety of CsA. These findings suggest that 0.1% CsA cationic emulsion is an effective and well-tolerated therapeutic option for patients with advanced DED, particularly in middle-aged women, and may serve as a cornerstone for long-term management of chronic ocular surface inflammation.

## Figures and Tables

**Table 1:** Baseline characteristics of patients before initiating 0.1% cyclosporine A therapy.

Baseline Characteristics of Study Participants Before Cyclosporine A Therapy Mean Values; ± Standard Deviation			
**Parameter**	**Mean ± SD**		
Age (years)	53.5; ±13.49		
Gender (female: male)	16:4		
OSDI Score (points)	53.13; ±13.78		
Oxford Scale (grade)	3.40; ±0.94		
	**Right Eye**	**Left Eye**	***p***
TBUT Test (s)	5.95; ±2.93	5.95; ±2.78	0.816
Schirmer Test (mm/5 min)	6.20; ±2.17	6.25; ±2.79	0.776
BCVA (Snellen)	0.84; ±0.26	0.82; ±0.2	0.556
Intraocular Pressure (mmHg)	16.75; ±3.23	16.65; ±3.17	0.876

No statistically significant differences were observed between the right and left eyes (*p* > 0.05).

**Table 2:** Changes in best-corrected visual acuity (BCVA) in the right and left eyes during 24 months of treatment with 0.1% cyclosporine A in patients with severe dry eye disease.

Eye	Time Point	Median	Min–Max	W (Shapiro–Wilk)	*p*
Right Eye	Before CsA	0.90	0.10–1.00	0.61	<0.001
	1–3 months	0.90	0.04–1.00	0.61	<0.001
	6 months	1.00	0.02–1.00	0.58	<0.001
	12 months	1.00	0.02–1.00	0.58	<0.001
	24 months	0.90	0.02–1.00	0.61	<0.001
Left Eye	Before CsA	0.90	0.40–1.00	0.82	0.002
	1–3 months	1.00	0.30–1.00	0.73	<0.001
	6 months	1.00	0.30–1.00	0.73	<0.001
	12 months	0.90	0.50–1.00	0.73	<0.001
	24 months	0.90	0.50–1.00	0.73	<0.001

**Table 3:** ANOVA results for intraocular pressure during therapy.

Eye	Time Point	IOP (Mean ± SD)	F	df	*p*	η^2^p
Right eye	Before CsA	16.75 ± 3.23	0.56	2.91, 55.31	0.637	0.03
	1–3 months	16.30 ± 3.63				
	6 months	16.35 ± 3.38				
	12 months	16.60 ± 2.84				
	24 months	16.90 ± 3.48				
Left eye	Before CsA	16.65 ± 3.17	0.31	2.72, 51.58	0.802	0.02
	1–3 months	16.45 ± 3.80				
	6 months	16.60 ± 3.24				
	12 months	16.95 ± 2.95				
	24 months	16.55 ± 3.22				

## Data Availability

The original contributions presented in this study are included in the article. Further inquiries can be directed to the corresponding author.

## Abbreviations

The following abbreviations are used in this manuscript:

CsA	Cyclosporine A
DED	Dry Eye Disease
SD	Standard Deviation
BCVA	Best Corrected Visual Acuity
OSDI	Ocular Surface Disease Index
TBUT	Tear Breakup Time
CFS	Corneal Fluorescein Staining
IOP	Intraocular Pressure
DEWS	Dry Eye Workshop
TFOS	Tear Film and Ocular Surface Society
M	Mean
F	F-value
Df	Degrees of Freedom
η^2^p	Partial Eta Squared
